# Is It Time for Treatment as Prevention of Chronic Hepatitis B?

**DOI:** 10.3390/pathogens12091137

**Published:** 2023-09-06

**Authors:** Jose A. Perez-Molina, Marta Rosas Cancio-Suárez, Santiago Moreno

**Affiliations:** 1 National Reference Centre for Tropical Diseases, Hospital Universitario Ramón y Cajal, 28034 Madrid, Spain; 2 IRYCIS, Carretera de Colmenar Km 9.1, 28034 Madrid, Spain; mrcancio.3@gmail.com (M.R.C.-S.); smoreno.hrc@salud.madrid.org (S.M.); 3 CIBER de Enfermedades Infecciosas, Instituto de Salud Carlos III, 28029 Madrid, Spain; 4 Infectious Diseases Department, Hospital Universitario Ramón y Cajal, 28034 Madrid, Spain; 5 Department of Medicine, Universidad de Alcalá, Alcalá de Henares, 28805 Madrid, Spain

**Keywords:** HBV, treatment, pre-exposure prophylaxis, pregnancy, nucleosides, vaccine

## Abstract

Hepatitis B is a major global health problem with high morbidity and mortality. Approximately 296 million people are living with chronic HBV, and 1.5 million new infections are detected each year, even though a highly effective vaccine has been available for decades and viral replication and transmission can be contained with the use of drugs. Nucleoside therapy, while not curative in most cases, can control viral replication, improve prognosis, and prevent mother-to-child transmission safely. Current treatment guidelines do not include a significant number of chronically infected patients or pregnant women and are often complex to implement. Since these populations continue to have a detectable HVB viral load, they could perpetuate transmission. Expanding and facilitating treatment indications, including treatment as a public health intervention, could help control the spread of the HBV pandemic, thus bringing us closer to the goal of the United Nations General Assembly for the year 2030.

## 1. Introduction

Hepatitis B is a major global health problem with high morbidity and mortality secondary to end-stage liver disease and hepatocellular carcinoma that, until recently, has largely been ignored as a health and development priority. An estimated 296 million people were living with chronic HBV infection (CHBI) worldwide in 2019, and 1.5 million new infections are detected each year [1], even though a highly effective vaccine has been available for decades and drug treatment, while not curative in most cases, can contain viral replication and transmission.

Perinatal transmission is the main risk factor for CHBI, which becomes chronic in 90% of cases. This rate increases from 20% to 50% for infections acquired between the ages of one and five years, compared to 5% for infections acquired in adulthood [1,2]. Mother-to-child transmission (MTCT); horizontal transmission (exposure to infected blood), especially from an infected child to an uninfected child during the first 5 years of life; and the percutaneous route are the most frequent modes of transmission for high- and intermediate-prevalence countries, while sexual relations and intravenous drug use are the most common for low-prevalence countries [1].

The control and eradication of viral hepatitis was set as the goal for the year 2030 by the United Nations General Assembly in 2015 under the Agenda for Sustainable Development Goals (Goal 3·3) [3] and, subsequently, by the World Health Assembly in 2016 under the Global Health Sector Strategy on Viral Hepatitis [4]. The viral hepatitis pandemic was responsible for an estimated 887,000 deaths in 2021 [1]; this figure is comparable to that reported for HIV, with 650,000 related deaths in the same year [5]. Without a comprehensive and determined response, the number of people living with HBV will remain high for the next 40–50 years and there will be a cumulative 20 million deaths between 2015 and 2030 [4].

## 2. Vaccination Is an Essential but Currently Insufficient Strategy

The Global Health Sector Strategy on Viral Hepatitis includes impact and service coverage targets aimed at eradicating hepatitis B by 2030 [4]. The impact goals include a 90% reduction in the incidence of new cases of CHBI and a 65% reduction in mortality, and the service coverage targets include childhood vaccine coverage, with three doses, of 90%; an HBV birth-dose vaccination coverage or other approaches to prevent MTCT of 90%; diagnosis of 90% of chronically infected people; and treatment of 80% of eligible persons with chronic HBV infection. Nevertheless, it is estimated that only 5% of 94 million persons eligible for treatment in 2016 [6] and 22% in 2019 [1] (i.e., those with a viral load > 20,000 IU/mL, increased transaminases, or cirrhosis) received antiviral therapy, and that less than 1% of mothers with a high viral load had received antiviral treatment to reduce MTCT [6]. In addition, only 13% of infants born to HBsAg-positive mothers received hepatitis B immunoglobulin (HBIG), along with a timely birth dose and follow-up vaccination [6].

Vaccination against HBV is a key strategy for controlling and potentially eradicating the infection. Its administration at birth impacts the population at the highest risk of chronic infection and prevents subsequent transmission. Nonetheless, we are still far from the proposed 2030 targets. In 2021, it was estimated that, globally, only 42% of newborns were vaccinated against HBV within 24 h of birth, and that only 80% of these received coverage with all three doses [7]. In addition, access to vaccination differed significantly by region, with the birth dose rate ranging from 17% for the African region to 78% for the Western Pacific; and from 71% to 91% for full three-dose rates between the African region and the European region, respectively [7].

HBV vaccines induce protective immunity in more than 90% of individuals, thus conferring long-term protection for at least 30 years [8]. However, efficacy may be lower in some populations, such as people living with HIV infection, people on haemodialysis, transplant recipients, people with chronic diseases, and people receiving immunosuppressive treatment [9]. In addition, given that anti-HBs levels may decline significantly over time in immunocompetent individuals, booster doses are increasingly recommended for at-risk populations, as the potential for lifelong immunity is unclear [9]. For these reasons, measures that complement vaccination are a necessary part of the hepatitis B eradication strategy.

## 3. Treatment of HBV Infection as a Complementary Strategy for Prevention

HIV and chronic HBV infection share many similarities as preventive strategies (Table 1), with the main difference being the lack of a vaccine to prevent HIV infection. However, there have been significant advances in the prevention of HIV infection through the control of viral replication using antiretroviral therapy. This strategy, known as “treatment as prevention” (TasP), has proven to be highly effective in preventing transmission through sexual relations and MTCT [10]. In fact, the term “undetectable = untransmittable” has been coined as a demonstration of the efficacy of TasP as a tool for controlling the HIV epidemic [11].

As for the HBV epidemic, antiviral treatment could play a more significant role in achieving eradication targets by 2030. This would require expansion of the current indications for treatment, which are more focused on individual health, so that they include wider populations, thus making it easier to achieve the set objectives for control and prevention of HBV infection.

## 4. Treatment of Chronically Infected Individuals beyond Established Indications

International guidelines on the management of CHBI recommend therapy in order to decrease the risk of liver-related complications, decrease the need for liver transplantation, restrict the development of hepatocellular carcinoma, and improve survival and quality of life [14,15,16]. However, apart from preventing MTCT, there is no mention of the prophylactic role of treating viraemic individuals in preventing transmission of HBV. Even new initiatives aimed at simplifying treatment algorithms fail to consider the preventive role of treatment outside pregnancy [17].

Hepatitis B is transmitted efficiently via sexual relations. In men who have sex with men (MSM), HBV transmission rates have been reported to be 8.6 times higher than HIV transmission rates, even though the prevalence of HBV infection in the study population was three times lower than that of HIV [18]. Unvaccinated MSM and heterosexual persons who have multiple sex partners or contact with sex workers are at particularly high risk [19]. It is estimated that in the USA, during 2013–2018, 38.2% of acute HBV infections were attributable to sexual transmission, and 12.6% of the 817,000 people aged 15 years and older living with CHBI had acquired the disease via sexual relations [20]. In Europe, acute cases mostly involved heterosexual transmission (30%), followed by transmission between MSM (16%) [21]. Migrants from areas with a high prevalence of CHBI are a particularly vulnerable population, representing 40% of new diagnoses of HBV infection in Europe (chronic in 93%) [21]. This mostly young and sexually active population is characterised by high infection rates and low vaccination coverage [22,23]. Difficulties accessing the health system because of legal barriers, stigmatisation, and cultural/language differences impair screening, vaccination, treatment, and counselling, thus favouring transmission [24].

Unsafe use of intravenous substances is the second leading cause of HBV infection in high-income countries [23,25]. Percutaneous exposure is very high-risk for a number of reasons: viral loads below 200 IU/mL in samples from patients with chronic HBV infection can infect chimpanzees [26]; the lowest documented HBV DNA level at which transmission has been reported is 200 IU/mL [27]; and ten or fewer viral particles are sufficient to start a readily detectable HBV infection if injected intravenously [28]. Consequently, guidelines for management of HBV-infected healthcare workers performing exposure-prone procedures recommend practice restrictions, or treatment of HBV when the viral load is not below the limit of detection or at least <200 IU/mL (CDC recommendation < 1000 IU/mL) [16,29,30], thus highlighting the high risk of transmission via percutaneous exposure.

The best strategy for preventing household transmission is early identification of people with chronic HBV infection, and vaccination of cohabitants [31]. By the same token, treatment of infected persons may be beneficial for unresponsive or unvaccinated cohabitants, especially in the case of immunosuppressed persons, in whom the risk of chronic disease is higher and the vaccine response rate lower.

Although no studies have explored the role of treatment as prevention in patients with CHBI, this strategy would be advisable considering the high transmissibility of the virus, transmission via less evident routes such as households, the benefits of early treatment of CHBI for disease progression, and experience from the field of HIV infection. Treatment could be proposed for all sexually active persons or intravenous drug users with an HBV viral load > 200–2000 IU/mL, regardless of age and other serological markers. The low-range threshold would include healthcare workers, intravenous drug users, and cohabitants of unvaccinated persons or non-responders. In addition, simplifying treatment indications could facilitate the implementation of guidelines in real-world settings, the current complexity of which is a barrier to treatment for eligible patients and contributes to health disparities in HBV care [17].

The potential drawbacks of increasing the number of candidates for treatment of CHBI include drug costs, problems with long-term adherence, toxicity, and the development of resistance. With regard to the issue of cost, tenofovir disoproxil fumarate (TDF) has been available as a generic drug for many years, with the result that its price has fallen significantly, thus making it affordable for large-scale treatment. The incidence of TDF-induced renal and bone toxicity is very low, especially in young people without predisposing factors. Increases in serum creatinine have been reported in up to 5% of patients after ten years of treatment, although only 2% were over 0.5 mg/dL [32,33]. Tenofovir alafenamide (TAF) has a better safety profile than TDF and could therefore be considered in high-risk cases, depending on the specific scenario [32,33]. On the other hand, toxicity monitoring would not involve substantial additional testing compared with an untreated CHBI patient, who requires continuous assessment of their CHBI status and of the need for antiviral treatment. The development of resistance in patients taking TDF is extremely rare, with only a few sporadic cases reported. Nevertheless, TDF-induced resistance-associated mutations are not well-characterised [32,33], and early initiation of treatment would only bring forward an option that will be introduced sooner or later in most cases.

## 5. Pre-Exposure Prophylaxis for HBV

In contrast to HIV infection, pre-exposure prophylaxis would play only a residual role in the prevention of HBV, owing to the availability of an effective vaccine. However, in people at high risk of exposure and no response to vaccination, or persons who do not wish to be vaccinated, the prophylactic use of TDF or TDF plus emtricitabine could be considered. In this regard, it has been reported that treatment or pre-exposure prophylaxis of HIV with antiretrovirals active against HBV can prevent HBV infection [34,35].

## 6. Pregnant Women

The basis of the strategy for preventing MTCT and infection during the first year of life is timely vaccination plus HBIG. No breakthrough infections have been reported in this scenario when the maternal HBV DNA viral load is ≤200,000 IU/mL, although a 4% to 30% transmission rate is described when the viral load is >200,000 IU/mL [36,37]. The addition of an effective drug with a high resistance barrier such as TDF during the third trimester further reduces the risk of transmission (OR 0.16; 95% CI: 0.10–0.26), especially when the DNA viral load is high [36]. Thus, international guidelines recommend prophylactic treatment of CHBI with TDF in pregnant women during the third trimester (starting at 24 to 28 weeks of gestation according to the EASLD guidelines) if their HBV DNA viral load is ≥200,000 IU/mL or HBV-eAg is positive (when their DNA viral load is unavailable) [14,16,36]. For women of childbearing age without advanced fibrosis who are planning to become pregnant in the near future, some guidelines even consider starting treatment when the child is born [16]. 

In countries where HBV is highly endemic, access to complete vaccination in the first year of life and to HBIG is limited. HBIG is a blood product that must be tested for infectious diseases. In addition, it is expensive, requires a cold chain, and may be in short supply. Moreover, rates as low as 17% for birth doses of HBV vaccine have been reported in the African region [7], and only 13% of infants born to HBsAg-positive mothers received HBIG with a timely birth dose and follow-up vaccination [6]. In addition, given that the diagnosis and follow-up of maternal HBV infection is hampered by access to serological diagnosis and determination of viral load, compliance with WHO recommendations is expected to be low [6,7].

The drop in DNA viral load after initiation of treatment during the third trimester could be insufficient, especially in cases of high viral load (≥6.3 log IU/mL) [36,37], when treatment is delayed, in the case of preterm delivery, and if invasive procedures are needed [38]. Up to 10%16% of foetal infections occur in utero [39]. It has been shown that median time to suppression of the viral load after initiation of treatment for hepatitis B is around six months [40], and that in pregnant women who started treatment with TDF in the third trimester, only 68% had HBV DNA < 200,000 IU/mL at delivery [37]. Consequently, a stricter HBV viral load threshold (4.0 log_10_ IU/mL) has been recommended before initiation of treatment [41]. There is increasing evidence that earlier initiation of treatment (during the first and second trimesters) provides better control of HBV viraemia during pregnancy and better maternal outcomes [42]. Furthermore, when TDF is initiated at gestational weeks 14–16 in highly viraemic mothers with CHBI, the rate of MTCT was shown to be 0% in infants who received the HBV vaccine but not HBIG [43]. Expanding the indications for TDF during the first trimester (or immediately after conception) could be a strategy for reducing MTCT and simplifying the management of CHBI in this scenario.

One of the most critical concerns for not starting HBV treatment during pregnancy is the safety of nucleoside analogue inhibitors. However, TDF has been shown to be safe in HIV-infected pregnant women from as early as conception and is considered a component of the preferred regimens during pregnancy. There is no evidence of human teratogenicity or increased risk of adverse pregnancy outcomes, preterm delivery, or infant mortality associated with this agent [13]. The safety profile for TAF looks similar. On the other hand, the impact of maternal TDF use on infant bone mineral status, while not established, remains uncertain and requires further longitudinal evaluation [13]. In pregnant women with CHBI but not HIV infection, there is solid evidence of the safety of TDF in the third trimester, and evidence of safety during the first and second trimesters is growing [13,36,41,42].

## 7. Conclusions

Hepatitis B is a global health problem that requires a determined response to prevent an additional 20 million deaths by 2030. Although vaccines and drugs that control viral replication are available, the challenges involved in their implementation are significant, as demonstrated by how far we have yet to go to achieve the 2030 Sustainable Development Goals. It is necessary to raise awareness of hepatitis B among all sectors of society, especially those responsible for developing guidelines and health policies.

A successful programme for broader implementation of HBV treatment must identify barriers such as cultural beliefs, stigma and treatment accessibility that could affect treatment uptake in the population, community and political stakeholder engagement, affordable access to medication, and long-term sustainability of programmes. Such initiatives can mirror those undertaken with the HIV infection, leading to near-universal access to antiretroviral drugs for treatment and prevention in the highest-prevalence countries.

Expanding treatment indications could help to control the spread of the HBV pandemic at different levels of intervention. Furthermore, there is growing evidence of the benefits of earlier treatment of CHBI for lowering the risk of hepatocellular carcinoma, reducing transcriptionally active integrations, inducing a slow decline in the cccDNA pool, and avoiding active disease [33]. Chronic therapy with nucleosides is safe and inexpensive.

In the long term, it is hoped that better drugs tailored to the needs of individual patients will become available, as has been the case in the field of HIV infection, with the ultimate goal of a cure for CHBI.

## 8. Highlights

HBV remains a global health issue despite vaccines and drugs therapy. We can curb the HBV pandemic by expanding our treatment options, namely:

- Early treatment for pregnant women;
- Treating all those with >200–2000 IU/mL, especially high-risk groups;
- Pre-exposure prophylaxis for vaccine non-responders and high-risk individuals.

## Figures and Tables

**Table 1:** Prevention strategies for HBV and HIV infection.

Prevention Strategy	HBV Infection	HIV Infection
Avoidance of exposure [1,5]	Use of condoms with sexual partners Avoidance of direct contact with blood and body fluids Not sharing sharp items such as razors, nail clippers, toothbrushes, and earrings or body rings Use of new or sterile needles for ear- or body-piercing, tattoos, and acupuncture Use of soap and water to cleanse wounds and skin sites that have been in contact with blood or body fluids Not donating blood, organs, or sperm	The same as for HBV Effective antiretroviral treatment prevents sexual transmission
Pre-exposure prophylaxis [1,6,7,12]	Immunisation with HBV vaccine with or without HBIG Immunisation with HBV vaccine of household and sexual contacts of people with chronic HBV infection Oral pre-exposure prophylaxis with TDF may play a role in patients who do not respond to HBV vaccine and are at high risk of exposure	Use of oral or injectable antiretrovirals and vaginal rings as pre-exposure prophylaxis
Mother-to-child transmission [1,6,13,14,15,16]	Treatment with TDF is recommended for pregnant women with HBV-DNA > 200,000 IU/mL starting at 28–32 weeks’ gestation Treatment with TDF starting at diagnosis of HBV or at the end of the first trimester Vaccine and HBIG for the newborn within 12–24 h of delivery	Treatment with antiretrovirals of all pregnant women to control replication of HIV
Treatment as prevention [12]	Expanding nucleoside treatment indications to all patients with >200–2000 IU/mL, especially for groups with a high risk of transmission (sexually active persons with partners of unknown vaccination status and IVDU)	Effective antiretroviral treatment prevents sexual transmission
Post-exposure prophylaxis [1,6,7,12,14,15,16]	Immunisation with HBV vaccine with or without HBIG, ideally within the first 24 h after exposure (this can be delayed 7–14 days depending on the route of infection).	Antiretroviral treatment for 28 days, starting within the first 72 h after exposure

The items shown in red are proposed preventive actions to complement current standard recommendations. IVDU: intravenous drug users; HBV: hepatitis B virus; HBIG: hepatitis B immunoglobulin; TDF: tenofovir disoproxil fumarate.
